# Metagenomic Next-Generation Sequencing for Diagnosis of Infectious Encephalitis and Meningitis: A Large, Prospective Case Series of 213 Patients

**DOI:** 10.3389/fcimb.2020.00088

**Published:** 2020-03-05

**Authors:** Xiao-Wei Xing, Jia-Tang Zhang, Yu-Bao Ma, Mian-Wang He, Guo-En Yao, Wei Wang, Xiao-Kun Qi, Xiao-Yan Chen, Lei Wu, Xiao-Lin Wang, Yong-Hua Huang, Juan Du, Hong-Fen Wang, Rong-Fei Wang, Fei Yang, Sheng-Yuan Yu

**Affiliations:** ^1^Department of Neurology, Hainan Hospital of PLA General Hospital, Sanya, China; ^2^Department of Neurology, First Medical Center of PLA General Hospital, Beijing, China; ^3^Medical School of Chinese PLA, Beijing, China; ^4^Department of Neurology, Fourth Medical Center of PLA General Hospital, Beijing, China; ^5^Department of Neurology, Eighth Medical Center of PLA General Hospital, Beijing, China; ^6^Department of Neurology, Sixth Medical Center of PLA General Hospital, Beijing, China; ^7^Department of Neurology, Seventh Medical Center of PLA General Hospital, Beijing, China; ^8^Department of Neurology, PLA Strategic Support Force Characteristic Medical Center, Beijing, China

**Keywords:** encephalitis, meningitis, pathogens, metagenomic next-generation sequencing, diagnosis

## Abstract

**Purpose:** We assessed the performance of metagenomic next-generation sequencing (mNGS) in the diagnosis of infectious encephalitis and meningitis.

**Methods:** This was a prospective multicenter study. Cerebrospinal fluid samples from patients with viral encephalitis and/or meningitis, tuberculous meningitis, bacterial meningitis, fungal meningitis, and non-central nervous system (CNS) infections were subjected to mNGS.

**Results:** In total, 213 patients with infectious and non-infectious CNS diseases were finally enrolled from November 2016 to May 2019; the mNGS-positive detection rate of definite CNS infections was 57.0%. At a species-specific read number (SSRN) ≥2, mNGS performance in the diagnosis of definite viral encephalitis and/or meningitis was optimal (area under the curve [AUC] = 0.659, 95% confidence interval [CI] = 0.566–0.751); the positivity rate was 42.6%. At a genus-specific read number ≥1, mNGS performance in the diagnosis of tuberculous meningitis (definite or probable) was optimal (AUC=0.619, 95% CI=0.516–0.721); the positivity rate was 27.3%. At SSRNs ≥5 or 10, the diagnostic performance was optimal for definite bacterial meningitis (AUC=0.846, 95% CI = 0.711–0.981); the sensitivity was 73.3%. The sensitivities of mNGS (at SSRN ≥2) in the diagnosis of cryptococcal meningitis and cerebral aspergillosis were 76.92 and 80%, respectively.

**Conclusion:** mNGS of cerebrospinal fluid effectively identifies pathogens causing infectious CNS diseases. mNGS should be used in conjunction with conventional microbiological testing.

**Trial Registration:** Chinese Clinical Trial Registry, ChiCTR1800020442.

## Introduction

Infectious encephalitis and meningitis are severe clinical conditions associated with high rates of morbidity and mortality worldwide (Venkatesan et al., 2013). However, the specific pathogens are not identified in >50% of patients with acute encephalitis cases (Glaser et al., 2006) because the volumes of available cerebrospinal fluid (CSF) may be low, and the blood-brain barrier causes pathogens to be retained in the brain. Conventional microbiological tests (smears, culture, immunological tests, and polymerase chain reaction) often fail to detect pathogens that cause encephalitis and meningitis. If early tests do not accurately identify the pathogen, treatment is often inappropriate, potentially triggering severe complications or death. Viral encephalitis and meningitis, tuberculous meningitis (TBM), bacterial meningitis, and fungal meningitis constitute most cases of infectious encephalitis and meningitis. However, these four types of infections may exhibit similar clinical manifestations and CSF findings (e.g., intracranial pressures, as well as white blood cell, glucose, and protein levels). Compared with the traditional method, there is lack of a reliable approach for the simultaneous identification of microorganisms, including various viruses, bacteria, fungi, etc.

Metagenomic next-generation sequencing (mNGS), a novel and promising approach, allows simultaneous and unbiased identification of all microorganisms in human samples (Goldberg et al., 2015; Forbes et al., 2017). Some studies have used mNGS to diagnose infectious central nervous system (CNS) diseases, but most of them included small sample sizes and were focused on specific viruses, bacteria, fungi or prokaryotes (Wilson et al., 2014; Guan et al., 2016; Yao et al., 2016; Xing et al., 2019). Recently, a somewhat larger study (58 patients) found that mNGS improved the diagnosis of neurological infections, but the mNGS-positivity rate was only 22% (Wilson et al., 2019). Thus, the performance of mNGS in diagnosis of infectious encephalitis and meningitis should be studied using a larger sample. Furthermore, because microbial genome sizes and lifestyles differ, interpretation of mNGS data requires careful analysis. Thus, we performed a large prospective study to evaluate mNGS performance in the diagnosis of infectious encephalitis and meningitis.

## Methods

### Study Design

**Figure 1:**
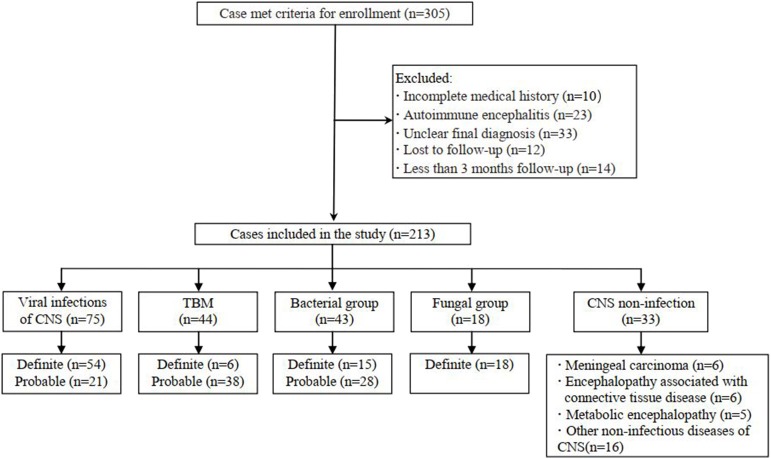
Flowchart of study participants.

This prospective multicenter study used clinical information to diagnose infectious encephalitis and/or meningitis in six teaching hospitals in Beijing, China. The inclusion criteria were as follows: high-level clinical suspicion of CNS infectious diseases; and lumbar puncture in uninfected patients. The exclusion criteria were as follows: refusal to undergo lumbar puncture; any contraindication for such puncture; and a diagnosis of autoimmune encephalitis. Eligible patients were divided into five groups according to their final diagnoses: viral encephalitis and/or meningitis, TBM, bacterial meningitis, fungal meningitis, and CNS non-infection. We included patients with definite or probable diagnoses of CNS viral infections (Jeffery et al., 1997), TBM (Marais et al., 2010) and bacterial meningitis (Mudaliar et al., 2006). Fungal meningitis was confirmed via conventional microbiological testing. Cryptococcal meningitis was confirmed via India ink staining or fungal culture, as well as histopathological evidence of cerebral aspergillosis. All patients had undergone at least 3 months of follow-up. Figure 1 shows the flow chart of study enrolment.

### mNGS of CSF

CSF specimens were collected in accordance with standard aseptic procedures, snap frozen, stored at −20°C, and subjected to mNGS within 24 h. The DNA of blood samples collected from healthy volunteers was fragmented and mixed with water in a certain proportions (negative controls). Glass beads were added to CSF samples, followed by vigorous agitation; DNA extraction; and DNA library construction. Quality-controlled libraries were sequenced on a BGISEQ-500/50 platform (BGI-Tianjin, Tianjin, China); an average of 20 million reads was obtained for each sample. After removal of human sequences, the remaining data were aligned to bacterial, virus, fungal and protozoan databases, as described in detail elsewhere (Xing et al., 2018, 2019).

### Interpretation of mNGS Data

A final sequencing list of suspected pathogenic microorganisms was obtained after removal of common background microorganisms and those that had appeared in >50% of samples during the past 3 months, compared to the negative controls. Next, the sequencing data list was analyzed in terms of species-specific read number (SSRN), genome coverage (%), and depth. Instead of SSRN, genus-specific read number was used for the *Mycobacterium tuberculosis* complex, because *M. tuberculosis* complex members exhibit >99.99% genomic sequence similarity (Supply and Brosch, 2017). Probable causative microorganisms were identified with reference to the literature and when the pathogenicity was consistent with the clinical manifestations; they were verified using traditional methods (serological tests, smears, cultures, and/or polymerase chain reaction assay). Herpes simplex virus (HSV), varicella zoster virus, Epstein–Barr virus, and cytomegalovirus were considered to be mNGS false-positives in patients with non-viral infections. Common pathogens of bacterial meningitis (*Streptococcus pneumoniae, Staphylococcus aureus, Haemophilus influenzae, Klebsiella pneumoniae*, and *Neisseria meningitidis*) were considered to be mNGS false-positives in patients with non-bacterial meningitis. *Aspergillus fumigatus, Aspergillus flavus, Aspergillus niger*, and *Aspergillus terreus* (Supply and Brosch, 2017) were considered to be mNGS false-positives in patients who did not have CNS aspergillosis.

### Statistical Analysis

Continuous data were considered to be nonparametric. Quantitative variables are expressed as medians (ranges) and qualitative variables are expressed as percentages. We drew receiver operating characteristic curves to compare diagnostic tests; test accuracies were represented by the area under the curve (AUC), such that a larger area implied a better test. Data processing was performed using SPSS Statistics version 21.0 (IBM Corp., Armonk, NY, USA).

## Results

### Patient Characteristics

In total, 213 patients treated in six hospitals were finally enrolled, including 75 with presumed viral encephalitis and/or meningitis, 44 with presumed TBM, 43 with presumed bacterial meningitis, 18 with fungal meningitis, and 33 with presumed non-infectious CNS diseases (Figure 1). All patients with infectious encephalitis and/or meningitis were screened by a single investigator (X.-W. X.) to determine diagnostic classification. Most patients (178/213, 83.57%) were treated in the First Medical Center of the PLA General Hospital. Non-infectious CNS conditions included meningeal carcinoma (6/33, 18%), encephalopathy associated with connective tissue disease (6/33, 18.18%), metabolic encephalopathy (5/33, 15.15%), and other non-infectious diseases (16/33, 48.48%). The clinical data are summarized in Table 1.

**Table 1:** Demographic and clinical characteristics of the 213 patients.

**Demographic and clinical characteristics**	**Viral infections of CNS** **(*n* = 75)**	**TBM (*n* = 44)**	**Bacterial meningitis** **(*n* = 43)**	**Fungal meningitis** **(*n* = 18)**	**CNS non-infection** **(*n* = 33)**	***P*-value**
Age, year	30 (14–64)	41 (14–76)	44 (14–76)	54.5 (15–80)	41 (14–72)	*p* < 0.001
Male no.	46 (61.33%)	25 (56.82%)	29 (67.44%)	13 (72.22%)	23 (69.70%)	*p* > 0.05
Time from onset to CSF collection, day	12 (2–441)	35 (11–790)	17 (1–201)	64 (6–351)	50 (5–714)	*p* < 0.001
Fever	72 (96.00%)	43 (97.73%)	41 (95.35%)	14 (77.78%)	20 (60.61%)	*p* < 0.001
Headache	60 (85.71%)	36 (92.31%)	35 (92.11%)	22 (91.67%)	19 (63.33%)	*p* < 0.01
Neck stiffness	46 (65.71%)	34 (87.18%)	31 (81.58%)	11 (45.83%)	7 (23.33%)	*p* < 0.001
Pressure, mmH_2_O	210 (100–550)	240 (110–500)	295 (120–400)	275 (75–330)	195 (80–330)	*p* < 0.01
WBC, × 10^6^/L	76 (0–720)	180 (4–2500)	1020 (1–68400)	95 (0–530)	8 (0–1131)	*p* < 0.001
Glucose, mmol/L	3.4 (0.9–5.5)	2.1 (0.4–5.55)	1.12 (0.01–3.7)	1.565 (0.58–5.0)	3.1 (1.2–5.9)	*p* < 0.001
Protein, g/L	0.775 (0.1–4.76)	1.65 (0.191–10)	1.914 (0.166–5.618)	1.189 (0.2–3.9)	0.652 (0.15–2.49)	*p* < 0.05

*CSF, cerebrospinal fluid; CNS, central nervous system; WBC, white blood cell; TBM, tuberculous meningitis*.

### Use of mNGS to Diagnose Viral Encephalitis and/or Meningitis

**Figure 2:**
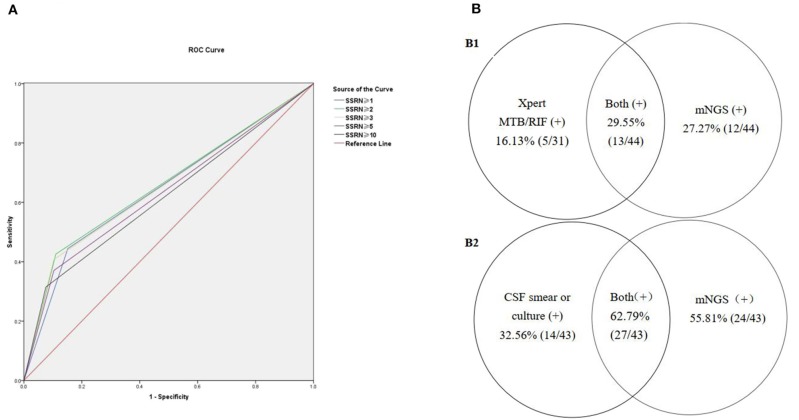
The positive rates of mNGS combined with conventional gold microbiologic testing. **(A)** Receiver operating characteristic (ROC) curves for diagnosing defined viral encephalitis by mNGS. **(B)** Comparison of pathogens detected by mNGS compared to conventional gold standard.

Seventy-five patients with viral encephalitis and/or meningitis (54 definite and 21 probable) were enrolled. An mNGS result was considered positive if the CSF sample exhibited SSRNs ≥1, 2, 3, 5, or 10; the diagnostic probability consistencies for viral encephalitis and/or meningitis were 32, 30.7, 29.3, 26.7, and 22.7%, respectively. The negative consistency rates were 84.8, 89.1, 89.9, 89.9, and 92.8%, respectively; the total consistency rates were 66.2, 68.5, 68.5, 67.6, and 68.1%, respectively. The AUCs for the five positive mNGS criteria were 0.584, 0.599, 0.596, 0.583, and 0.577, respectively. When an SSRN ≥2 was considered positive, the corresponding AUC (0.599) was relatively larger than when other SSRNs were considered positive. For the 54 patients with definite viral encephalitis and/or meningitis, if an SSRN ≥2 was considered positive, the AUC was largest (0.659, 95% confidence interval [CI] = 0.566–0.751; Figure 2A); the positive, negative, and total consistency rates were 42.6, 89.1, and 76.0%, respectively (Table 2, Table S1).

**Table 2:** Comparison of the diagnostic efficacy of five positive mNGS criteria for viral encephalitis and/or meningitis (Definite, *n* = 54).

**SSRN**	**Positive consistent rate**	**Negative consistent rate**	**Total consistent rate**	**AUC**	**SE**	**95% CI**
≥1	0.444	0.848	0.734	0.646	0.047	0.554–0.738
≥2	0.426	0.891	0.760	0.659	0.047	0.566–0.751
≥3	0.407	0.899	0.760	0.653	0.047	0.560–0.746
≥5	0.370	0.899	0.760	0.634	0.048	0.541–0.728
≥10	0.315	0.928	0.755	0.621	0.048	0.527–0.715

*SSRN, species-specific read number; AUC, area under the curve; SE, standard error; CI, confidence interval*.

### Use of mNGS to Diagnose TBM

Forty-four patients were diagnosed with TBM (six definite and 38 probable). An mNGS result was considered positive if the CSF samples exhibited genus-specific read numbers ≥1, 2, 3, 5, and 10; the associated positive consistency rates were 27.3, 20.5, 18.2, 13.6, and 6.8%, respectively. The negative consistency rates were 96.4, 97.6, 98.2, 99.4, and 100%, respectively; the total consistency rates were 82.2, 81.7, 81.7, 81.2, and 80.8%, respectively. When a genus-specific read number ≥1 was considered positive, the AUC (0.619, 95% CI = 0.516–0.721) was largest. Among the six patients with definite TBM (including five who were positive on Xpert MTB/RIF and one who was positive on acid-fast staining of nerve tissue), the sensitivity and specificity of mNGS were 66.67% (4/6) and 96.45% (163/169), respectively. Among the 44 patients with presumed TBM (six definite and 39 probable), the CSF Xpert MTB/RIF positivity rate was 16.13% (5/31) and the mNGS positivity rate was 27.27% (12/44) (Table S2); the combined mNGS and Xpert MTB/RIF positivity rate was 29.55% (13/44) (Figure 2B1). Unfortunately, acid-fast bacillus staining and *M. tuberculosis* culture results were negative for all CSF samples.

### Use of mNGS to Diagnose Bacterial Meningitis

Forty-three patients were diagnosed with bacterial meningitis (15 definite and 28 probable). An mNGS result was considered positive if the SSRNs were ≥2, 3, 5, 10, and 15; the positive consistency rates were 62.8, 62.8, 55.8, 55.8, and 51.2%, respectively. The negative consistency rates were 82.9, 87.6, 95.9, 95.9, and 95.9%, respectively; total consistency rates were 78.9, 82.6, 87.8, 87.8, and 86.9%, respectively. The AUCs for the five positive mNGS criteria were 0.729, 0.752, 0.758, 0.758, and 0.735, respectively. When SSRNs ≥5 or 10 were considered positive, the AUC (0.758, 95% CI = 0.663–0.854) was largest. The sensitivity, specificity, positive predictive value, and negative predictive value of mNGS in the diagnosis of 15 patients with definite bacterial meningitis were 73.3, 95.9, 61.1, and 97.6%, respectively (Table S3) and the AUC was largest (0.846, 95% CI = 0.711–0.981). In brief, among the 43 patients with presumed bacterial meningitis (15 definite and 28 probable), mNGS identified a bacterial pathogen in 24 (55.8%, 24/43); conversely, the CSF combined Gram stain/culture-positive rate was only 32.56% (14/43). Combination of the two methods increased the positive rate to 62.79% (27/43) (Figure 2B2). In the 24 patients with positive mNGS results (SSRNs ≥5 or 10), the top three pathogens were *S. pneumoniae* (41.7%, 10/24), *K. pneumoniae* (12.5%, 3/24), and *Listeria monocytogenes* (8.3%, 2/24). Of note, among the patients with non-bacterial meningitis, 4.1% (7/170) were false-positive on mNGS for *S. aureus* (57.1%, 4/7), *S. pneumoniae* (28.6%, 2/7), and *H. influenzae* (14.3%, 1/7).

### Use of mNGS to Diagnose Fungal Meningitis

Among the 13 patients with definite cryptococcal meningitis, the first-time-positive rates of CSF India ink staining and fungal culture were 0 and 38.4%, respectively. After multiple stainings/cultures, the positive rate of the above two methods were 84.62%. Cryptococcal antigen was detected in all (12/12) CSF samples. When an SSRN ≥2 was considered mNGS-positive, the sensitivity, specificity, positive predictive value, and negative predictive value were 76.92% (10/13), 99.52% (1/207), 90.91% (10/11), and 98.56% (206/209), respectively (Table S4). In addition, among the 13 patients, five were co-infected with *Cryptococcus neoformans* sensu lato and *Cryptococcus gattii* sensu lato, as has been described in detail elsewhere (Xing et al., 2019). Among the five patients with confirmed cerebral aspergillosis, when an SSRN ≥2 was considered mNGS-positive, the sensitivity, specificity, positive predictive value, and negative predictive value were 80% (4/5), 79.3% (165/208), 8.5% (4/47), and 99.40% (165/166), respectively (Table S5).

### Use of mNGS to Diagnose Four Types of CNS Infection

The mNGS-positive rate for diagnosis of the four types of definite or probable infectious encephalitis and meningitis, including viral encephalitis and/or meningitis, TBM, bacterial meningitis, and fungal meningitis, was 40.6% (73/180) (Table 3). The positivity rate for the four types of definite CNS infectious diseases was 57.0% (52/93).

**Table 3:** Positive mNGS results of the four types of CNS infection (73/180).

**Results of mNGS**				**Confirmatory data**
**Pathogen**	**SSRN**	**Coverage, %**	**Depth**	
**Viral encephalitis and/or meningitis (*****n*** **=** **23), SSRN** **≥** **2**				
Herpes simplex virus 1 (*n* = 9)	2,764 (2–27639)	57.9066 (0.0412–91)	2.99 (1–7.5)	PCR (*n* = 5), Positive HSV antibody (CLIA) (*n* = 9)
Herpes simplex virus 2 (*n* = 1)	4,311	90	6.5	Positive HSV antibody (CLIA)
Varicella-zoster virus (*n* = 5)	77 (6–3626)	4.3204 (0.2258–95.6227)	1 (1–2.47)	Skin herpes zoster (*n* = 4), PCR (*n* = 1)
Epstein-Barr virus (*n* = 6)	124.5 (4–3807)	6.8258 (0.1022–71.6965)	1 (1–2.39)	PCR (*n* = 3); Neuropathology (*n* = 1, DLBCL); Positive EBV antibody (*n* = 4)
Cytomegalovirus (*n* = 1)	4	0.081	1	CSF cytomegalovirus IgG (122.0 U/mL)
Human adenovirus B1 (*n* = 1)	2,467	62.309	9.84	Clinical evidence
**Tuberculous meningitis (*****n*** **=** **12), GSRN** **≥** **1**				
*Mycobacterium tuberculosis* complex	4 (1–1046)	ND	ND	Xpert MTB/RIF (*n* = 3); A. TB or T-SPOT.TB (*n* = 4); Tuberculosis antibody (*n* = 1); Clinical evidence (*n* = 4)
**Bacterial meningitis (*****n*** **=** **24), SSRN** **≥** **5 or 10**				
*Streptococcus pneumoniae* (*n* = 10)	2,488 (19–34711)	6.0112 (0.2236–66.8601)	1.31 (1–4.56)	Smear/culture (*n* = 4),
*Streptococcus pyogenes* (*n* = 1)	453	3.7	1	Smear
*Streptococcus intermedius* (*n* = 1)	592	5.2651	1	Clinical evidence
*Klebsiella pneumoniae* (*n* = 3)	15 (12–70)	0.0241 (0.0127–0.2979)	1 (1–1.59)	Culture (*n* = 3)
*Listeria monocytogenes* (*n* = 2)	43.5 (36–51)	0.1167 (0.0418–0.1915)	1.01 (1–1.02)	Culture (*n* = 2)
*Nocardia farcinica* (*n* = 1)	277	0.2631	1.16	Clinical evidence
*Brucella* (*n* = 1)	18 (GSRN)	ND	ND	RBPT(+) and SAT(+)
*Stenotrophomonas maltophilia* (*n* = 1)	288	0.7879	1	Clinical evidence
*Haemophilus influenzae* (*n* = 1)	12	0.1478	1	Clinical evidence
*Escherichia coli* (*n* = 1)	58	1.2399	1	Culture
*Aggregatibacter aphrophilus* (*n* = 1)	256	0.7625	4.61	Clinical evidence
*Neisseria meningitidis* (*n* = 1)	4543	44.2621	1.82	Clinical evidence
**Fungal meningitis (*****n*** **=** **14), SSRN or GSRN≥2**				
*C. neoformans s.l*. (*n* = 10)	40.5 (2–177203)	0.01445 (0.0019–71)	1 (1–24)	India ink staining or culture for fungi
*C. gattii s.l*. (*n* = 5)	334 (7–71743)	0.1885 (0.0136–20)	1.02 (1–8.7)	India ink staining or culture for fungi
*Aspergillus* (*n* = 4)	6 (3–9) (GSRN)	ND	ND	Histopathology

*CLIA, chemiluminescence immunoassay; CNS, central nerve system; DLBCL, diffused large b-cell lymphoma; GSRN, genus-specific reads number; mNGS, metagenomic next-generation sequencing; PCR, polymerase chain reaction; RBPT, rose bengal plate agglutination test; SAT, serum agglutination test; s.l., sensu lato; SSRN, species-specific read number*.

*Positive HSV antibody: a 4-fold or more increase in CSF virus-specific antibody titer, or CSF virus-specific IgM antibody*.

*Positive EBV antibody: VCA (viral capsid antigens)-IgG ≥1:640, EA (early antigens)-IgG ≥1: 160, or EBNA (EB nuclear antigens) ≥1: 2; [Proposed Guidelines for Diagnosing Chronic Active Epstein-Barr Virus Infection]*.

*Clinical evidence: patient's history, clinical presentation, imaging finding, routine laboratory CSF results and response to antibiotic treatment*.

## Discussion

We explored whether mNGS, combined with conventional microbiological testing, aided in the diagnosis of infectious encephalitis and meningitis. All patients met the criteria for definite or probable diagnosis of CNS infection. We made two important observations. First, different CNS infections were associated with different positive diagnostic criteria because of variations in genomic sequences and lifestyles. Second, mNGS is more effective for detection of CNS infections, compared to conventional methods (Figure 2B). Furthermore, mNGS combined with conventional microbiological testing improved detection of CNS infections.

Among the 75 patients with presumed encephalitis and/or meningitis (54 definite and 21 probable), all pathogens identified via mNGS were DNA viruses (mostly HSV-1, HSV-2, and varicella zoster virus), consistent with the findings of other studies (Steiner et al., 2007; Tyler, 2018). However, tumors and HSV infection can both trigger autoimmune encephalitis (Pruss et al., 2012). Thus, in the viral encephalitis and/or meningitis group, all patients with suspected autoimmune disorders were excluded. Identification of specific viral encephalitis/meningitis pathogens is difficult; the gold standard pathogen-specific polymerase chain reaction assays fail to identify many viral families that infect the CNS (Koyuncu et al., 2013). In theory, mNGS is useful for detection of all pathogens in clinical samples. We found that mNGS did not significantly predict viral encephalitis and/or meningitis (0.5 < AUC < 0.7), possibly attributable in part to the absence of RNA detection. RNA viruses, such as enteroviruses and Japanese encephalitis virus, are common causes of viral encephalitis and meningitis (Le et al., 2010; Ai et al., 2017). Thus, DNA/RNA co-extraction methods must be improved and DNA and RNA sequenced simultaneously to improve virus detection rates.

There were 10 million new cases of tuberculosis worldwide in 2017, of which 558,000 were rifampicin-resistant. Although only 1% of all tuberculosis infections involve the CNS, TBM is the most serious manifestation of TB; over 50% of affected patients become disabled or die (Thwaites et al., 2013). However, early accurate diagnosis of TBM remains challenging, which greatly affects patient outcomes. The pathogen complex is termed *M. tuberculosis* complex; *M. tuberculosis, Mycobacterium africanum, Mycobacterium bovis*, and *Mycobacterium canettii* were all detected in the present study. Thus, mNGS is of great utility in terms of TBM diagnosis when at least one specific read is matched to the *M. tuberculosis* complex, consistent with the findings of previous studies (Miao et al., 2018; Wang et al., 2019). Although the diagnostic utility of mNGS for TBM is not high (0.619), mNGS is undoubtedly very useful given the current diagnostic predicament. Of note is that the specificity of mNGS in the diagnosis of tuberculous meningitis is 96.4%, which allows a negative mNGS test to be used as one of the diagnostic methods to exclude TBM.

Bacterial meningitis is a considerable burden worldwide, associated with high-level morbidity, mortality, and disability (Chaudhuri, 2004). *S. pneumoniae, N. meningitidis, H. influenzae*, and *L. monocytogenes* are the most common pathogens of community-acquired suppurative meningitis (Bijlsma et al., 2016). We found that *S. pneumoniae* was the principal causative pathogen in this study. When SSRNs ≥5 or 10 were considered positive, mNGS performance was optimal in terms of bacterial meningitis diagnosis (AUC = 0.846); mNGS may be very useful in this context. However, *S. aureus* is a very common pathogen that may cause a false-positive result if present in CSF.

We found that that the diagnostic utility of mNGS for cryptococcal meningitis was poorer than the utility of traditional methods (India ink staining, culture, and cryptococcal antigen detection). The thick cryptococcal capsule may not have been adequately breached, and DNA could not exit. Cell wall breakage must be improved. We found that five patients had been infected with *C. neoformans* s.l. and *C. gattii* s.l., and thus required prolonged courses of antifungal therapy (Perfect et al., 2010; Chen et al., 2013). mNGS of CSF can be used to identify *Cryptococcus* species, facilitating cryptococcal meningitis diagnosis and management. It is difficult to identify the source of cerebral aspergillosis; histopathology is the gold standard diagnostic method (Bao et al., 2014). Although there were few patients with cerebral aspergillosis in this study, we presume that mNGS may serve as a future, frontline diagnostic test for cerebral aspergillosis because of the non-invasive nature of mNGS.

We analyzed the diagnostic utility of mNGS in patients with four common forms of infectious encephalitis and/or meningitis; we speculate that mNGS could be used in diagnosis of all infectious CNS diseases, including those caused by rare or new pathogens. mNGS was better than conventional methods in the diagnosis of infectious encephalitis and/or meningitis, especially in terms of species identification. However, this novel approach should be used in conjunction with conventional microbiological testing. The data should be interpreted differently, depending on the pathogen involved.

Our study had some strength. The principal strength was that we divided eligible patients into groups with different CNS infections, rather than pooling all patients with CNS infections. The mNGS results were interpreted with reference to the different types of infections. Moreover, the study was prospective in nature. However, there were also several limitations in this study. Firstly, RNA-Seq data were not tested in parallel with DNA sequencing, which might provide valuable complementary information. Furthermore, because DNA extraction efficiency is critical in terms of mNGS results, a comparison of the extraction efficiencies of the various kits must be performed in future studies. Finally, our sample size was relatively small, especially after stratification of patients according to the types of infections.

As a novel form of microbiological testing, mNGS affords certain advantages over traditional tests when identifying pathogens causing infectious encephalitis and meningitis. The new technology exhibits great potential. Careful attention is needed with respect to DNA and RNA co-extraction methods, extraction efficiency, differentiation of colonization from infection, and method standardization (Kennedy et al., 2017; Simner et al., 2018).

## Data Availability Statement

The raw data supporting the conclusions of this article will be made available by the authors, without undue reservation, to any qualified researcher. Some of sequencing data is available at: http://www.ncbi.nlm.nih.gov/bioproject/PRJNA544609.

## Ethics Statement

This study was approved by the Ethics Committee of the Chinese PLA General Hospital (approval no. S2018-198-01). Written informed consent was obtained from all patients or their legal representatives.

## Author Contributions

J-TZ designed the study. S-YY organized and delivered the expert meeting, which was attended by all the authors. Y-BM, M-WH, G-EY, WW, X-KQ, X-YC, LW, X-LW, Y-HH, JD, H-FW, R-FW, and FY contributed to acquisition of clinical data. X-WX conducted analyses and wrote the manuscript. All of the authors read and approved the final article.

### Conflict of Interest

The authors declare that the research was conducted in the absence of any commercial or financial relationships that could be construed as a potential conflict of interest.
