# Supplementary material for: Metagenomic Next-Generation Sequencing for Diagnosis of Infectious Encephalitis and Meningitis: A Large, Prospective Case Series of 213 Patients
Source: Front Cell Infect Microbiol. 2020 Mar 5;10:88. doi: 10.3389/fcimb.2020.00088 (PMC7066979; doi:10.3389/fcimb.2020.00088)
Supplement: Supplementary file 1 [file Data_Sheet_1.docx]

**Supplementary material |** Diagnostic Performance of mNGS Compared with Clinical Testing.

| **Table S1** | | | |
| --- | --- | --- | --- |
| SSRN≥2 | Definite | | |
|  | VE/VM | Non-VE/VM | |
| mNGS(+) | 23 | 15 | |
| mNGS(-) | 31 | 123 | |
| Positive consistent rate | | | 42.6% |
| Negative consistent rate | | | 89.1% |
| Total coincidence rates | | | 76% |

| **Table S2** | | | |
| --- | --- | --- | --- |
| GSRN≥1 | Definite and probable | | |
|  | TBM | Non-TBM | |
| mNGS(+) | 12 | 6 | |
| mNGS(-) | 32 | 163 | |
| Positive consistent rate | | | 27.3% |
| Negative consistent rate | | | 96.4% |
| Total coincidence rates | | | 82.2% |

| **Table S3** | | | |
| --- | --- | --- | --- |
| SSRN≥5 or 10 | Definite | | |
|  | BM | | Non-BM |
| mNGS(+) | 11 | | 7 |
| mNGS(-) | 4 | | 163 |
| Sensitivity | | 73.3% | |
| Specificity | | 95.9% | |
| PPV | | 61.1% | |
| NPV | | 97.6% | |

| **Table S4** | | |
| --- | --- | --- |
| SSRN≥2 | Definite | |
|  | CM | Non-CM |
| mNGS(+) | 10 | 1 |
| mNGS(-) | 3 | 206 |
| Sensitivity | 76.92% | |
| Specificity | 99.52% | |
| PPV | 90.91% | |
| NPV | 98.56% | |

| **Table S5** | | |
| --- | --- | --- |
| SSRN≥2 | Definite | |
|  | CA | Non-CA |
| mNGS(+) | 4 | 43 |
| mNGS(-) | 1 | 165 |
| Sensitivity | 80% | |
| Specificity | 79.3% | |
| PPV | 8.5% | |
| NPV | 99.40% | |

Abbreviations: BM, bacterial meningitis; CM, cryptococcal meningitis; CA, cerebral aspergillosis; mNGS, metagenomic next-generation sequencing; NCT, negative consistent rate; NPV, negative predictive value; PCT, positive consistent rate; PPV, positive predictive value; TBM, tuberculous meningitis; TCR, total coincidence rates; VE/VM, viral encephalitis and viral meningitis.
